# The Legacy of the First Valve: Outcomes of Redo Surgical Aortic Valve Replacement After Prior Transcatheter Versus Prior Surgical Aortic Valve Replacement—A Narrative Review

**DOI:** 10.3390/jcm15103640

**Published:** 2026-05-09

**Authors:** Dimitrios E. Magouliotis, Serge Sicouri, Vasiliki Androutsopoulou, Andrew Xanthopoulos, Vanesa Brecher, Massimo Baudo, Basel Ramlawi

**Affiliations:** 1 Department of Cardiac Surgery Research, Lankenau Institute for Medical Research, Wynnewood, PA 19096, USA; sicouris@mlhs.org (S.S.); vb4075@pcom.edu (V.B.); massimo.baudo@icloud.com (M.B.); ramlawib@mlhs.org (B.R.); 2 Department of Cardiothoracic Surgery, Faculty of Medicine, University of Thessaly, Biopolis, 41110 Larissa, Greece; androutsopoulouvasiliki@uth.gr; 3 Department of Cardiology, Faculty of Medicine, University of Thessaly, Biopolis, 41110 Larissa, Greece; andrewvxanth@gmail.com; 4 Department of Cardiac Surgery, Lankenau Medical Center, Wynnewood, PA 19096, USA

**Keywords:** transcatheter aortic valve replacement, surgical aortic valve replacement, reoperation, lifetime management, aortic stenosis, valve durability, propensity score matching

## Abstract

Transcatheter Aortic Valve Replacement (TAVR) has transformed aortic stenosis management across the full risk spectrum, but expansion into younger populations makes valve failure and reintervention central to lifetime planning. There are two pathways to follow when TAVR fails: redo transcatheter implantation and surgical explantation with surgical aortic valve replacement (SAVR), termed TAVR-SAVR. This narrative review synthesizes evidence from four studies (35,677 patients, 2011–2024) examining the association between prior valve type and redo SAVR outcomes versus redo SAVR after prior surgical prosthesis (SAVR-SAVR). TAVR-SAVR volume grew at up to 144.6% annually, projected to surpass SAVR-SAVR by approximately 2029 based on linear extrapolation from limited registry and single-center data. Operative mortality was 12.3–17% in TAVR-SAVR versus 1.1–9% in SAVR-SAVR, persisting after propensity matching in both comparative studies (11.3% vs. 6.7%, OR 1.7; and 12.0% vs. 1.1%, OR 12.5). Observed-to-expected mortality ratios exceeded 1.0 across all risk strata, including low-risk patients (O/E up to 5.48), while SAVR-SAVR demonstrated a remarkably low ratio of 0.22–0.33. Renal failure, failure to rescue, and prolonged ventilation were significantly higher following TAVR-SAVR; stroke and pacemaker rates were comparable. Paradoxically, shorter bypass and cross-clamp times in TAVR-SAVR despite worse outcomes are consistent with cumulative organ injury, rather than operative complexity, as a predominant contributor to excess mortality, though this mechanistic explanation remains hypothetical. STS risk models, developed for standard surgical populations, showed limited applicability in this population, with observed mortality consistently exceeding predicted values. These findings raise important considerations regarding TAVR-first strategies in operable patients aged 65–80 years, although causality cannot be established from observational data alone. In the era of expanding TAVR indications, the legacy of the first valve cannot be ignored.

## 1. Introduction

Aortic stenosis is the most prevalent valvular heart disease in the developed world, affecting more than 5% of adults over the age of 65 years and carrying a poor prognosis once symptomatic if left untreated [1]. Over the past two decades, transcatheter aortic valve replacement (TAVR) has fundamentally transformed its management. Initially approved in 2011 for patients deemed inoperable or at prohibitive surgical risk, TAVR has since demonstrated non-inferiority and, in some analyses, superiority to surgical aortic valve replacement (SAVR) across the full spectrum of surgical risk, culminating in its approval for low-risk patients in 2019 [2,3,4,5].

The consequence of this paradigm shift has been extraordinary. Annual TAVR volumes in the United States surpassed SAVR for the first time in 2019 and have continued to grow at an accelerating rate, with more than 100,000 procedures performed annually in recent years [6]. Critically, this growth has been accompanied by a progressive decline in the mean age and surgical risk profile of TAVR recipients. Where TAVR was once the exclusive domain of octogenarians with multiple comorbidities, it is now increasingly offered to patients in their seventh decade of life with life expectancies potentially exceeding 20 years [7,8].

This demographic shift introduces a challenge that was largely irrelevant in the early TAVR era: prosthesis durability and the feasibility of valve reintervention. Transcatheter heart valves, like surgical bioprostheses, are subject to structural valve deterioration (SVD) over time, with clinically significant SVD expected to occur in a meaningful proportion of patients within 10 to 15 years of implantation [9,10]. For patients who undergo TAVR in their mid-sixties, this durability horizon falls squarely within their expected lifespan, making reintervention not a remote possibility but a near certainty for many [11].

When TAVR fails, two principal reintervention strategies exist. The first is redo transcatheter valve-in-valve implantation (ViV-TAVR), in which a second transcatheter valve is deployed within the failed prosthesis. The second is surgical explantation of the transcatheter valve with SAVR, referred to herein as TAVR-SAVR. Each pathway carries distinct risks and benefits. ViV-TAVR offers the advantage of avoiding sternotomy and carrying lower short-term procedural risk; however, it is constrained by anatomical limitations including coronary obstruction risk, residual hemodynamic gradients in small prostheses, and the accumulated complexity of multiple layers of valve material [12,13]. TAVR-SAVR, by contrast, provides definitive anatomic correction and potentially superior long-term durability through a new surgical bioprosthesis, but demands a technically demanding explantation procedure and carries the biological burden of an already compromised patient [14,15].

Against this background, a pivotal but incompletely answered question emerges: does prior TAVR affect the outcomes of subsequent SAVR, compared with redo SAVR performed in patients who previously received a surgical prosthesis (SAVR-SAVR)? The answer to this question has direct and immediate implications for the initial valve selection strategy in every patient candidate for both TAVR and SAVR, especially those aged 65 to 80 years, for whom current American College of Cardiology/American Heart Association (ACC/AHA) guidelines recommend shared decision-making between the two approaches [16].

This narrative review synthesizes the available comparative evidence addressing this question, examining temporal trends in TAVR-SAVR volume, operative outcomes stratified by prior valve type, risk model performance, and the mechanistic drivers of any observed mortality excess. The implications for lifetime valve management, patient counseling, and the reappraisal of TAVR-first strategies in operable patients are critically discussed.

Literature Search: A structured literature search was conducted across PubMed, Embase, and the Cochrane Library from inception through March 2026. Search terms included combinations of: “transcatheter aortic valve replacement”, “surgical aortic valve replacement”, “TAVR explant”, “redo SAVR”, “reoperation”, and “reintervention”. Studies were eligible for inclusion if they reported operative outcomes of SAVR performed after prior aortic valve replacement, included at least 50 patients undergoing SAVR after prior TAVR, and reported operative mortality. Case reports, editorials, and conference abstracts were excluded. Four studies met these criteria and form the evidence base of this review.

## 2. The Growing Burden of TAVR Reintervention

The expansion of TAVR to younger and lower-risk patient populations has set in motion a cascade of long-term consequences that were not anticipated during the technology’s early adoption. The fundamental biological reality is that all bioprosthetic valves, either SAVR or TAVR, are subject to progressive structural deterioration over time [17,18]. Calcium deposition, leaflet thickening, and fibrotic remodeling collectively reduce valve area and increase transvalvular gradients, ultimately resulting in clinically significant SVD in a proportion of patients that grows with each passing year after implantation [19].

The durability of transcatheter heart valves has been incompletely characterized, principally because the technology’s rapid adoption outpaced the generation of long-term follow-up data. Ten-year results from the Nordic Aortic Valve Intervention (NOTION) trial and the PARTNER 1 trial have provided the most mature data currently available, demonstrating SVD rates of approximately 14% to 28% at 8 to 10 years in TAVR cohorts [20,21]. These figures, while informative, are derived from predominantly elderly, high-risk populations with significant competing mortality risk. In younger, lower-risk patients who are expected to survive well beyond 10 years, the actuarial burden of SVD is expected to be substantially higher [22].

Beyond SVD, TAVR valves may fail through several additional mechanisms. Paravalvular leak (PVL), which occurs due to incomplete sealing between the prosthesis frame and native annulus, remains more prevalent after TAVR than after SAVR and is associated with increased mortality even at mild severity [23]. Prosthetic valve endocarditis (PVE) complicates approximately 0.5% to 1.5% of TAVR cases annually, with TAVR-PVE carrying a mortality rate exceeding 40% when managed medically and with particularly challenging surgical outcomes [24,25]. Device malposition, coronary obstruction, and valve thrombosis constitute additional failure mechanisms that may mandate urgent surgical intervention [26].

The aggregate consequence of these failure mechanisms is a rapidly growing population of patients requiring reintervention after TAVR. Bowdish and colleagues, analyzing the Society of Thoracic Surgeons (STS) Adult Cardiac Surgery Database between 2012 and 2023, documented a 4235% overall increase in cardiac surgery after TAVR, with TAVR explant and SAVR representing the fastest-growing subcategory with an average annual growth rate of 148.1% [27]. Fukuhara and colleagues, reporting on the institutional experience at the University of Michigan, demonstrated that TAVR-SAVR cases increased linearly at a rate of 4% per year as a proportion of all aortic valve reoperations, from 0% in 2011 to 31.3% in 2024, with projections suggesting that TAVR-SAVR will surpass SAVR-SAVR in annual volume by 2029 [28], although this projection is based on linear extrapolation from a single institutional series and should be interpreted with appropriate caution.

The profile of patients requiring TAVR-SAVR has also shifted substantially over time. Early series, drawn from the high-risk TAVR cohorts of 2011 to 2015, consisted predominantly of elderly patients with multiple comorbidities in whom urgent or emergent reoperation was required for early device failure [29]. More recent series increasingly include low- and intermediate-risk patients who underwent elective TAVR during the expansion era, returning for SVD or PVE years after implantation. Fukuhara and colleagues reported that low-risk patients began appearing in their TAVR-SAVR cohort in 2018 and surpassed high-risk patients in annual volume by 2021 [30]. This demographic evolution has important implications: while these patients carry lower predicted operative risk, their observed-to-expected (O/E) mortality ratios after TAVR-SAVR are disproportionately elevated, a phenomenon discussed in detail in Section 5.

The ViV-TAVR alternative to surgical explantation is not universally applicable. Anatomical constraints including coronary height, LVOT obstruction risk, and prosthesis design preclude ViV-TAVR in a substantial proportion of patients with failed TAVR valves [31]. Furthermore, even when technically feasible, ViV-TAVR may produce residual hemodynamic impairment due to the constrained internal dimensions of the original prosthesis, particularly in smaller annuli [32]. Long-term durability data for ViV-TAVR are limited, and the accumulating leaflet material from successive transcatheter implants may accelerate subsequent valve deterioration. These constraints mean that a meaningful proportion of patients with failed TAVR will have no viable transcatheter reintervention option and must be offered surgical explantation [12,33].

## 3. Comparative Evidence: Study Characteristics

Four principal studies have addressed the comparative outcomes of redo SAVR stratified by prior valve type, collectively encompassing 35,677 patients across a combined observation period of 2011 to 2024. These studies vary substantially in design, sample size, data source, and time period, collectively providing a layered perspective on an evolving clinical problem. The key characteristics of each included study are summarized in Table 1.

### 3.1. Early Descriptive Evidence from the STS Database: Jawitz et al., 2020 [29]

The first systematic examination of SAVR after TAVR outcomes was published by Jawitz and colleagues in 2020, representing an analysis of the STS Adult Cardiac Surgery Database spanning July 2011 to March 2015 [29]. The study identified 123 patients who underwent SAVR following prior TAVR during this period, representing approximately 0.3% of the estimated 40,000 TAVR procedures performed in the United States during the same window. This was a purely descriptive cohort study with no SAVR-SAVR comparator arm; its primary analytical contribution was the calculation of observed-to-expected (O/E) mortality ratios, stratified by preoperative surgical risk category.

The study population was predominantly elderly (median age 77 years) and high-risk, with 59% classified as having an STS Predicted Risk of Mortality (STS-PROM) greater than 8% at the time of reoperation. The most common indications for reoperation included PVL (15.5%), SVD (11.4%), failed repair (10.6%), sizing and positioning issues (10.6%), and PVE (9.8%). Overall operative mortality was 17.1%, substantially exceeding the 4.6% reported for contemporary redo SAVR after prior surgical bioprosthesis in matched cohorts [35]. Procedural complexity was reflected in a median operative time of 321 min, more than 100 min longer than published benchmarks for primary SAVR.

### 3.2. Landmark Comparative Analysis of TAVR-SAVR Versus SAVR-SAVR Using the STS Database: Hawkins and Fukuhara et al., 2023 [34]

The first comparative study to directly examine TAVR-SAVR against SAVR-SAVR was published by Hawkins, Fukuhara, and colleagues in 2023, representing the landmark analysis in this field [34]. Data were extracted from the STS Database spanning 2011 to 2021, identifying 31,106 patients undergoing bioprosthetic SAVR after prior aortic valve procedure. The cohort was stratified into three groups: 1126 patients with prior TAVR (TAVR-SAVR), 674 patients with prior SAVR and subsequent TAVR (SAVR-TAVR-SAVR), and 29,306 patients with prior SAVR (SAVR-SAVR).

Baseline characteristics demonstrated substantial differences between groups. TAVR-SAVR patients were older (median age 74 vs. 67 years), more frequently classified as having prior heart failure (73% vs. 57%), and carried higher rates of insulin-dependent diabetes, chronic lung disease, end-stage renal disease, and cerebrovascular disease. Elective surgical status was notably less frequent in TAVR-SAVR patients (34% vs. 57%), reflecting a greater burden of acute or urgent presentations. Active endocarditis was present in 28% of TAVR-SAVR patients, compared with 20% of SAVR-SAVR patients.

Risk adjustment was performed using hierarchical logistic regression incorporating STS-PROM as a fixed effect and hospital as a random effect. A subanalysis restricted to isolated SAVR cases (excluding concomitant procedures other than CABG) was performed with propensity score matching using 63 preoperative variables, yielding 433 well-matched pairs. This propensity-matched analysis is the primary comparative analysis referenced throughout this review, as it most effectively addresses the confounding imbalance between groups.

### 3.3. Trends and Risk Factors in Cardiac Surgery After TAVR: Bowdish et al., 2024 [27]

Bowdish and colleagues published a comprehensive analysis of cardiac surgery after TAVR using the STS Database from January 2012 to March 2023, identifying 5457 patients who underwent any cardiac operation after prior TAVR [27]. Of these, 2972 (54.5%) underwent SAVR with or without concomitant cardiac procedures, while the remaining 2485 patients (45.5%) underwent non-SAVR cardiac surgery. This study did not include a SAVR-SAVR comparator arm and is therefore classified as a descriptive analysis contributing trend, volume, and risk factor data rather than comparative outcome estimates.

The principal contributions of this study to the current synthesis are threefold. First, it documented the dramatic temporal increase in cardiac surgery after TAVR, rising 4235% overall and 144.6% per year over the study period, with TAVR explant and SAVR representing the fastest-growing subcategory. Second, it characterized the procedural complexity of TAVR-SAVR, noting that concomitant aortic root or sinus procedures were required in 28.8% of cases, with full root replacement necessary in 13.4%. Third, it identified independent predictors of operative mortality in TAVR-SAVR patients through multivariable logistic regression, finding that surgical urgency, age, dialysis, and concomitant CABG or mitral valve surgery, but notably not TAVR device type, were associated with increased mortality.

### 3.4. Refined Institutional Comparative Analysis of Isolated Redo SAVR After TAVR: Fukuhara et al., 2026 [28]

The most contemporary and methodologically refined comparative study was published by Fukuhara and colleagues in 2026, reporting the institutional experience at the University of Michigan between 2011 and 2024 [28]. The study identified 1024 consecutive patients undergoing redo SAVR, of whom 127 had prior TAVR (TAVR-SAVR) and 897 had prior bioprosthetic SAVR (SAVR-SAVR). Critically, the analysis was restricted to patients undergoing isolated SAVR with or without concomitant CABG (57 TAVR-SAVR and 447 SAVR-SAVR) in order to remove the confounding effect of highly variable concomitant procedure burden, a known limitation of the Hawkins 2023 analysis.

Risk adjustment was performed using propensity score matching incorporating 16 clinically relevant preoperative variables, yielding 50 TAVR-SAVR and 93 SAVR-SAVR patients with excellent baseline balance. Uniquely, this study utilized the newly developed STS SAVR-after-TAVR risk calculator for STS-PROM estimation in the TAVR-SAVR group, rather than the standard isolated SAVR model, providing more appropriate risk benchmarking for this population. The study also reported O/E mortality ratios stratified by the original risk profile at the time of index TAVR, offering a novel perspective on the evolution of risk across the lifetime valve management journey.

## 4. Unadjusted and Risk-Adjusted Outcomes

### 4.1. Operative Mortality

Operative mortality, defined as death within 30 days of surgery or prior to hospital discharge, is the primary outcome of interest across all included studies and represents the most consistent and compelling signal in the available evidence. Across the full spectrum of study designs, databases, and time periods represented in this review, TAVR-SAVR is uniformly and substantially associated with higher operative mortality than SAVR-SAVR.

In the Jawitz 2020 analysis, the earliest cohort of TAVR-SAVR patients reported in the literature, overall operative mortality was 17.1% [29]. This figure was striking in its magnitude: contemporary benchmarks for isolated redo SAVR after surgical bioprosthesis reported operative mortality rates of approximately 4.6% to 9%, suggesting a two- to four-fold excess risk associated with prior TAVR even in this early descriptive series [35]. The 17.1% figure was particularly alarming among low-risk patients, in whom an O/E mortality ratio of 5.48 was observed, indicating that actual deaths were more than five times higher than predicted by the STS risk model.

The Hawkins and Fukuhara 2023 STS database analysis provided the first comparative perspective, demonstrating unadjusted operative mortality of 17% in TAVR-SAVR versus 9% in SAVR-SAVR (*p* < 0.001) across the full cohort of 31,106 patients [34]. After hierarchical logistic regression adjusting for STS-PROM, operative mortality remained significantly higher for TAVR-SAVR compared with SAVR-SAVR (OR 1.53, 95% CI 1.14–2.06, *p* = 0.004). The analysis then applied propensity score matching to isolate the effect of prior valve type in patients undergoing isolated SAVR, yielding 433 well-matched pairs. In this matched cohort, operative mortality was 11.3% for TAVR-SAVR versus 6.7% for SAVR-SAVR (OR 1.74, 95% CI 1.09–2.86, *p* = 0.020), confirming that the mortality excess persisted even after controlling for 63 preoperative variables including comorbidity burden, functional status, operative urgency, and valvular pathology.

Bowdish and colleagues documented an overall operative mortality of 14.1% in their cohort of 2972 SAVR-after-TAVR patients, with procedure-specific O/E ratios consistently exceeding 1.0 across all STS validated procedure categories, ranging from 1.21 for CABG combined with mitral valve repair to 2.11 for isolated mitral valve replacement performed concurrently [27]. These elevated O/E ratios across procedure types confirm that the excess mortality observed in TAVR-SAVR patients is not restricted to a particular surgical combination but represents a broadly applicable risk amplification attributable to the prior transcatheter prosthesis.

The Fukuhara 2026 institutional analysis, restricted to the more homogeneous population of patients undergoing isolated SAVR with or without CABG, demonstrated the starkest mortality differential. In the pre-matching cohort, operative mortality was 12.3% for TAVR-SAVR versus 1.1% for SAVR-SAVR (*p* < 0.001), a more than 11-fold difference [28]. After propensity score matching, TAVR-SAVR operative mortality was 12.0% versus 1.1% for SAVR-SAVR (OR 12.5, *p* = 0.008). The magnitude of this OR, substantially larger than the 1.74 observed in the Hawkins 2023 analysis, likely reflects the institutional cohort’s methodological refinement: by restricting to isolated SAVR and applying contemporary matching incorporating the new SAVR-after-TAVR STS risk calculator, residual confounding from concomitant procedure burden was minimized, allowing a cleaner estimate of the true impact of prior valve type.

The trend in TAVR-SAVR operative mortality over time deserves specific attention. Fukuhara and colleagues reported a significant improvement in operative mortality across three eras: 27.3% in Era 1 (2013–2017), 7.4% in Era 2 (2018–2021), and 5.6% in Era 3 (2022–2024) (*p* = 0.049) [30]. This improvement likely reflects a combination of factors including growing institutional experience with TAVR explantation techniques, improved patient selection through multidisciplinary structural heart team evaluation, earlier surgical referral before advanced cardiorenal decompensation, and refinements in perioperative management. However, even in the most contemporary era, operative mortality remains substantially higher than the SAVR-SAVR benchmark of 1.1%, underscoring the persistent excess risk.

### 4.2. Failure to Rescue

Failure to rescue, defined as death following a major postoperative complication, is a quality-sensitive metric that reflects a system’s capacity to manage adverse events once they occur, rather than simply their incidence. This endpoint was reported by Hawkins and colleagues and represents one of the most clinically illuminating findings in the comparative literature [34].

In the propensity-matched cohort, the rate of major morbidity (defined by the STS composite of stroke, prolonged ventilation, deep sternal wound infection, reoperation, and renal failure) was not significantly different between TAVR-SAVR and SAVR-SAVR groups (28% vs. 24%, *p* = 0.223). However, among patients who developed a major complication, mortality was significantly higher in the TAVR-SAVR group: 32% versus 16% (*p* = 0.008), corresponding to an approximately two-fold higher failure-to-rescue rate.

This finding has profound mechanistic implications. It suggests that the excess operative mortality in TAVR-SAVR patients is not primarily driven by a higher incidence of complications, statistically comparable between groups, but rather by a diminished physiological reserve to survive complications once they occur. TAVR-SAVR patients, carrying a greater burden of heart failure, renal dysfunction, and multiorgan comorbidity accumulated over the years between their initial TAVR and subsequent reoperation, appear less capable of mounting the metabolic and cardiovascular response necessary to recover from major postoperative morbidity. This interpretation is supported by the observation that multiorgan failure, rather than isolated cardiac causes, accounted for the predominant mode of death in the Fukuhara 2026 series (85.7% of deaths in the TAVR-SAVR arm) [28].

### 4.3. Renal Failure

Renal failure was consistently more frequent following TAVR-SAVR than after SAVR-SAVR across the comparative studies. In the Hawkins 2023 propensity-matched cohort, acute renal failure occurred in 9% of TAVR-SAVR patients versus 5% of SAVR-SAVR patients (*p* = 0.043) [34]. In the Fukuhara 2026 pre-matching analysis, newly started dialysis was required in 9.4% of TAVR-SAVR versus 1.8% of SAVR-SAVR patients (*p* = 0.001), although this difference did not reach statistical significance in the smaller matched cohort (10.4% vs. 3.3%, *p* = 0.130) [28].

The excess renal failure burden in TAVR-SAVR patients is multifactorial. Preoperative renal impairment is substantially more prevalent in the TAVR-SAVR population. In fact, Fukuhara and colleagues reported creatinine levels of 1.2 versus 1.0 mg/dL (*p* < 0.001) in the pre-matching cohort, thus reflecting the cumulative renal consequences of heart failure, contrast exposure from catheter-based procedures, and nephrotoxic medications. Intraoperative factors including longer overall operative times, greater hemodynamic instability, and higher transfusion requirements may compound perioperative renal injury. The combination of preoperative renal vulnerability and intraoperative hemodynamic stress creates a substrate for acute-on-chronic kidney disease that significantly amplifies mortality risk when renal failure occurs in the perioperative period.

### 4.4. Prolonged Ventilation

Prolonged mechanical ventilation, defined as ventilation exceeding 24 h after surgery, is a marker of systemic physiological decompensation and a driver of length of stay, infectious complications, and mortality. In the Jawitz 2020 descriptive series, prolonged ventilation was required in 40.7% of TAVR-SAVR patients which is substantially higher than published benchmarks for redo SAVR [29].

In the comparative analysis by Hawkins and colleagues, prolonged ventilation rates in the propensity-matched cohort trended higher in TAVR-SAVR patients (22% vs. 19%), though the difference did not achieve statistical significance (*p* = 0.243) [34]. Fukuhara and colleagues, in their institutional series, reported significantly higher rates of prolonged ventilation in the pre-matching cohort (36.8% vs. 9.4%, *p* < 0.001) and in the propensity-matched cohort (32.0% vs. 9.7%, *p* < 0.001) [28]. The discrepancy between the two comparative studies likely reflects differences in cohort composition (the Fukuhara series restricted to isolated SAVR, removing the contribution of longer concomitant procedures) and institutional variability in extubation protocols.

### 4.5. Hospital Length of Stay

Hospital length of stay was consistently longer following TAVR-SAVR in both comparative studies. Hawkins and colleagues reported a postoperative length of stay of 8 days for TAVR-SAVR versus 7 days for SAVR-SAVR, though the difference did not reach statistical significance in the propensity-matched analysis (*p* = 0.351) [34]. Fukuhara and colleagues found a significant difference in length of stay in their matched cohort (9 vs. 7 days, *p* = 0.005), as well as significantly longer ICU stays (97 vs. 70 h in the pre-matching analysis) [28]. Extended hospital stays following TAVR-SAVR reflect the combined consequences of prolonged ventilation, greater hemodynamic instability, and the higher incidence of renal and respiratory complications described above. Beyond their direct resource implications, prolonged hospital admissions are independently associated with nosocomial infection, deconditioning, and adverse patient experience.

### 4.6. Stroke and Permanent Pacemaker Implantation—Null Findings

Not all outcomes were worse in TAVR-SAVR patients. Stroke rates were comparable between groups in both comparative studies: 4% versus 3% (*p* = 0.853) in the Hawkins 2023 matched cohort, and 4% versus 0% (*p* = 0.120) in the Fukuhara 2026 matched cohort [28,34]. Similarly, permanent pacemaker (PPM) implantation rates were not significantly different: 8.6% versus 7.1% (*p* = 0.790) in the Fukuhara matched analysis.

The absence of a stroke excess in TAVR-SAVR patients is counterintuitive given the additional embolic risk theoretically associated with TAVR valve explantation—manipulation of calcified, neo-endothelialized material with the potential to cause cerebral embolization. One possible explanation is that the higher prevalence of cerebrovascular disease at baseline in TAVR-SAVR patients introduces competing ascertainment challenges. Another possibility is that the shorter cardiopulmonary bypass times in TAVR-SAVR (discussed in Section 6) mitigate intraoperative stroke risk. The comparable PPM rates are perhaps less surprising: TAVR-SAVR patients have a substantially higher baseline prevalence of permanent pacemakers (33.3% vs. 5.6% in the Fukuhara pre-matching cohort), reflecting the pacemaker burden inherent to the TAVR procedure itself, and new postoperative PPM requirement at rates not dissimilar to SAVR-SAVR [28].

### 4.7. TAVR Device Type

A clinically important question in TAVR-SAVR outcomes is whether the type of implanted transcatheter prosthesis (balloon-expandable versus self-expanding) influences the risk of subsequent surgical explantation. Both device families present distinct technical challenges at explantation: balloon-expandable valves such as the SAPIEN family are mounted on a cobalt–chromium frame that becomes embedded within the aortic annulus through neo-endothelialization, while self-expanding devices such as the Evolut family involve a tall nitinol stent frame that extends into the LVOT and requires circumferential dissection for removal [36].

Despite these theoretical differences in technical difficulty, neither Bowdish 2024 nor Fukuhara 2026 found a significant association between TAVR device type and operative mortality [27,28]. In the Bowdish multivariable analysis, balloon-expandable versus self-expanding TAVR type was not independently associated with operative mortality (OR 0.925, *p* = 0.657). Fukuhara and colleagues similarly reported that valve type distribution (self-expanding 50.9%, balloon-expandable 45.6%, mechanically expandable 3.5%) did not influence outcomes in their matched analysis. These convergent null findings across two different datasets reinforce the conclusion that excess TAVR-SAVR mortality reflects patient-level biological factors rather than device-specific technical factors (Table 2).

## 5. The Observed-to-Expected Mortality Paradox

Among the most clinically consequential findings in the TAVR-SAVR literature is the consistent demonstration that existing risk prediction models (specifically the STS Predicted Risk of Mortality (STS-PROM) calculator) show limited applicability in the TAVR-SAVR population, with observed mortality consistently exceeding predicted values. It is important to note that the STS-PROM was not designed or validated for this population; some degree of miscalibration is therefore an expected consequence of applying a model outside its intended scope, rather than a reflection of fundamental model inadequacy. This finding, manifested as an O/E mortality ratio substantially greater than 1.0, has been documented across multiple studies, multiple time periods, and multiple risk strata. It has important implications for preoperative patient counseling, surgical decision-making, and the validity of risk-based arguments for TAVR in younger patients.

The STS-PROM model was developed and validated in surgical populations undergoing primary or redo SAVR after prior surgical prostheses. It incorporates preoperative variables such as age, sex, comorbidities, functional status, and operative urgency, but was not designed for the biological context of TAVR explantation [37]. Critically, it does not incorporate prior TAVR as a distinct risk variable, treating it as equivalent to prior surgical reoperation. This equivalence is incorrect. The presence of an embedded transcatheter prosthesis introduces additional surgical complexity, requires specific explantation maneuvers, and is associated with a biologically distinct patient-level risk profile shaped by cumulative organ injury accumulated during heart failure progression between TAVR implantation and reoperation.

The magnitude of STS-PROM underestimation was first quantified by Jawitz and colleagues, who demonstrated O/E ratios of 5.48 (95% CI 1.17–13.93) in low-risk patients (STS-PROM <4%), 1.66 (95% CI 0.35–4.40) in intermediate-risk patients, and 1.16 (95% CI 0.68–1.79) in high-risk patients [29]. The paradoxical finding that O/E underestimation is greatest in low-risk patients is mechanistically important. Low-risk patients underwent TAVR at a time when their surgical risk profile was genuinely low; however, the biological cascade initiated by heart failure progression, renal injury, and cumulative comorbidity accumulation over the years between TAVR and reoperation transforms them into a physiologically distinct and more vulnerable population than their preserved STS-PROM score suggests.

Bowdish and colleagues confirmed systematic O/E underestimation across all STS procedure categories in their 2024 analysis [27]. For isolated SAVR after TAVR, the O/E ratio was 1.42, indicating that observed mortality was 42% higher than predicted. For SAVR combined with concomitant procedures, O/E ratios were even more pronounced: 1.68 for CABG combined with AVR, 2.11 for isolated MVR performed at the time of TAVR explant, and 1.34 for CABG combined with MVR. These elevated O/E ratios across procedure types reinforce the conclusion that model underestimation is a property of the TAVR-SAVR patient population, not an artifact of any specific procedure combination.

Fukuhara and colleagues introduced a critical methodological refinement in their 2026 data analysis: application of the newly developed STS SAVR-after-TAVR risk calculator, which was specifically designed to estimate operative mortality in patients undergoing surgical reoperation after prior TAVR [28]. Using this calculator, the O/E ratio for TAVR-SAVR in their institutional cohort was 1.50 (95% CI 0.60–3.09), with subgroup O/E ratios of 1.80, 1.58, and 1.21 across low-, intermediate-, and high-risk categories, respectively. This indicates that the new calculator, while more accurate than its predecessor, continues to underestimate operative mortality in TAVR-SAVR patients, particularly at the lower end of the risk spectrum.

The contrasting picture in SAVR-SAVR patients is equally instructive. Fukuhara and colleagues reported a remarkably low O/E ratio of 0.33 (95% CI 0.11–0.76) in the full SAVR-SAVR cohort, declining further to 0.22 (95% CI 0–1.20) in the propensity-matched cohort [28]. An O/E ratio of 0.22–0.33 implies that observed mortality in SAVR-SAVR patients at the University of Michigan was less than one-third of the predicted mortality—a testimony to the safety of redo SAVR in patients with prior surgical prostheses at experienced high-volume centers. This benchmark establishes SAVR-SAVR not merely as a reference comparator but as an exceptionally safe procedure when performed at institutions with dedicated expertise in reoperative cardiac surgery.

The clinical implications of systematic O/E underestimation in TAVR-SAVR extend beyond individual patient counseling. At a health systems level, performance benchmarking using STS-PROM as the expected outcome measure will systematically penalize centers that accept the highest-risk TAVR-SAVR cases, potentially creating perverse incentives for surgical risk aversion in a patient population that urgently requires expert surgical care. The development and validation of accurate, TAVR-specific risk calculators is therefore not merely an academic exercise but a prerequisite for appropriate quality measurement and incentive alignment in the evolving era of TAVR reintervention [38,39].

**Figure 1:**
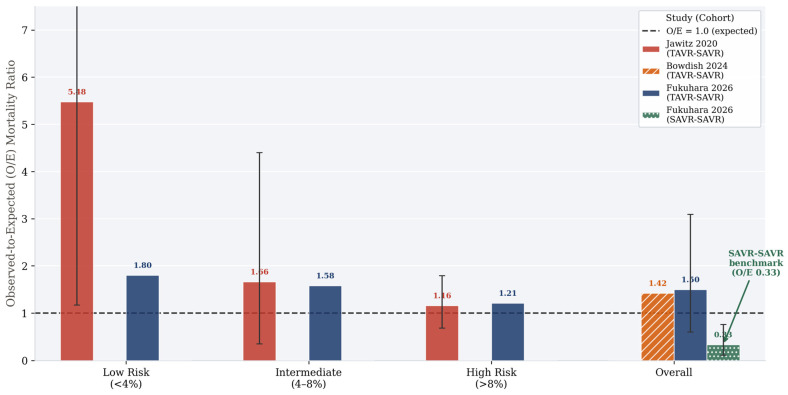
Observed-to-expected (O/E) operative mortality ratios in TAVR-SAVR patients stratified by preoperative surgical risk category across included studies. All risk categories demonstrate O/E ratios exceeding 1.0, indicating systematic underestimation of operative mortality by existing STS risk models. The strikingly elevated O/E ratio in low-risk patients (O/E 5.48) is particularly notable and directly relevant to the counseling of younger, lower-risk patients considering TAVR as their initial valve procedure. The SAVR-SAVR cohort O/E ratio of 0.22–0.33 is shown for reference, underscoring the safety of redo SAVR after prior surgical bioprosthesis at experienced centers. Error bars represent 95% confidence intervals where reported. Abbreviations: TAVR-SAVR = surgical aortic valve replacement after prior transcatheter aortic valve replacement; SAVR-SAVR = redo surgical aortic valve replacement after prior surgical aortic valve replacement; STS-PROM = Society of Thoracic Surgeons Predicted Risk of Mortality; O/E = observed-to-expected [27,28,29,34].

An additional dimension of the O/E paradox concerns risk stratification at the time of the initial TAVR procedure. Fukuhara and colleagues stratified TAVR-SAVR patients by their original risk profile at the time of index TAVR, reporting O/E ratios of 1.80 in patients who were low-risk at the time of TAVR, 1.92 in intermediate-risk patients, and 0.74 in high-risk patients [28]. The highest O/E ratios in the low- and intermediate-risk groups (precisely the patients for whom TAVR-first strategies are most actively debated) suggest that the biological transformation between TAVR implantation and subsequent reoperation disproportionately affects patients who initially appeared well-positioned to tolerate future reintervention. This finding directly challenges the assumption that choosing TAVR in a low-risk patient creates a favorable surgical safety net for future operations. The O/E mortality ratios across risk strata and included studies are summarized in Figure 1, illustrating the consistent and systematic underestimation of operative mortality by existing STS risk models in TAVR-SAVR patients across all risk categories, in stark contrast to the SAVR-SAVR benchmark (Table 3).

## 6. The Operative Complexity Paradox

One of the most intellectually provocative and clinically instructive observations in the TAVR-SAVR literature is a paradox that emerges when operative data are examined alongside outcomes: despite TAVR-SAVR patients experiencing significantly higher operative mortality, more frequent prolonged ventilation, greater renal failure burden, and worse overall outcomes, they undergo procedures with shorter cardiopulmonary bypass (CPB) times and shorter aortic cross-clamp times than their SAVR-SAVR counterparts.

In the Hawkins 2023 propensity-matched analysis, median CPB time was significantly shorter in TAVR-SAVR patients (113 vs. 133 min, *p* < 0.001), as was median cross-clamp time (83 vs. 96 min, *p* < 0.001) [34]. Fukuhara and colleagues similarly reported shorter CPB time (131 vs. 146 min, *p* = 0.020) and shorter cross-clamp time (92 vs. 114 min, *p* = 0.003) in the TAVR-SAVR group prior to matching, with concordant but non-significant trends in the matched cohort [28]. These shorter operative times in TAVR-SAVR patients are mechanistically plausible: a substantial proportion of TAVR-SAVR cases (61.2% in the Fukuhara series) were performed without prior sternotomy, as the original TAVR was performed percutaneously. Without the additional time required for redo sternotomy, including careful dissection of adhesions, pericardial entry, and cardiac decompression, the cardiac portion of the procedure can proceed more expeditiously.

Therefore, the paradox is that the group with shorter operative times and lower surgical complexity (as measured by CPB and cross-clamp duration) experienced substantially worse outcomes. In conventional cardiac surgical thinking, longer bypass times are associated with higher postoperative morbidity and mortality through mechanisms including systemic inflammatory activation, ischemia–reperfusion injury, hemodilution, and end-organ hypoperfusion. The reversal of this expected relationship in TAVR-SAVR versus SAVR-SAVR is therefore a powerful signal that operative complexity is not the primary driver of excess TAVR-SAVR mortality.

What, then, explains the excess mortality? Converging evidence from multiple studies points to patient-level biological factors (specifically, the cumulative organ injury that accumulates during the interval between TAVR implantation and surgical reoperation) as the predominant mechanism. By the time TAVR-SAVR patients reach the operating room, they have typically endured years of progressive heart failure. This commonly manifests as right ventricular dysfunction, pulmonary hypertension, and elevated filling pressures that persist despite aortic valve treatment [28]. Chronic low cardiac output compounds this trajectory through renal and hepatic consequences including reduced glomerular filtration, hepatic congestion, and impaired synthetic function. The result is a substrate of multiorgan vulnerability that amplifies the physiological demands of even a technically straightforward operation.

Fukuhara and colleagues proposed a specific mechanism they termed the “cardiorenal spiral”—a hypothetical framework in which patients with failing TAVR valves sustain more frequent and cumulative cardiac insults than their post-SAVR counterparts, as each episode of valve-related hemodynamic deterioration (whether from progressive SVD, intermittent PVL-related volume loading, or endocarditis-associated septic physiology) imposes additional stress on the right ventricle, pulmonary vasculature, and kidneys [28]. While this framework is clinically plausible and consistent with multiorgan failure as the predominant mode of death, it remains speculative in the absence of prospective longitudinal data and should be interpreted as a proposed mechanistic explanation rather than an established pathway. This cumulative multi-system injury is also compounded by a phenomenon of delayed surgical referral: patients and referring cardiologists, aware of the high operative risk associated with TAVR explantation, often delay surgical referral beyond the optimal therapeutic window, allowing progressive cardiorenal decompensation to reach a point at which even technically successful surgery fails to reverse the underlying biological crisis.

The observation that multiorgan failure, rather than primary cardiac arrest or neurological events, was the dominant cause of death in TAVR-SAVR patients (85.7% of deaths in the Fukuhara matched cohort) is entirely consistent with this cardiorenal injury model [28]. Multiorgan failure is the terminal common pathway of refractory hemodynamic instability compounded by renal, hepatic, and respiratory insufficiency, precisely the biological trajectory predicted by the cumulative organ injury hypothesis. The absence of a stroke excess and the comparable rates of primary cardiac causes of death between TAVR-SAVR and SAVR-SAVR further support the conclusion that the operative procedure itself is not the source of excess mortality.

The operative complexity paradox has a critical practical implication: it shifts the focus of intervention for improving TAVR-SAVR outcomes away from technical refinements in explantation technique, which are clearly important but may have limited mortality impact, toward earlier identification and referral of patients with failing TAVR valves, before cardiorenal decompensation becomes irreversible. Fukuhara and colleagues specifically identified ‘prolonged wait time before surgical referral, often due to reluctance to proceed with TAVR explant despite a failed valve’ as a key contributor to poor outcomes [28]. This reluctance paradoxically produces the outcomes it seeks to avoid. Delayed referral allows progressive organ injury that dramatically increases actual operative risk, regardless of the motivation behind the delay.

The phenomenon of SAVR-SAVR patients having longer CPB and cross-clamp times yet better outcomes provides further evidence that procedural duration per se is not the mortality driver. In SAVR-SAVR cases, longer bypass times typically reflect greater surgical complexity (e.g., redo sternotomy adhesiolysis, more involved aortic root procedures, or larger annular enlargements) performed in patients who, despite the procedural demands, retain the physiological reserve to tolerate extended cardiopulmonary support and mount a successful recovery. The contrast with TAVR-SAVR patients, who undergo shorter but biologically more demanding procedures, encapsulates the central message of this literature: in reoperative aortic valve surgery, it is the patient’s biology, not the surgeon’s technical task, that ultimately determines survival.

These insights argue for a fundamentally different approach to risk stratification and patient selection in TAVR-SAVR. Rather than focusing exclusively on preoperative STS-PROM scores, which systematically underestimate operative risk in this population, comprehensive multidimensional assessment incorporating right ventricular function, pulmonary artery pressures, nutritional status, frailty indices, and hepatorenal reserve may better characterize operative risk and identify patients who require urgent versus elective surgical referral [38].

## 7. Volume Trends and the 2029 Projection

The quantitative trajectory of TAVR-SAVR volume growth represents one of the most compelling arguments for treating this as a public health priority rather than a rare surgical curiosity. Across all four included studies (spanning the STS national database, a single institutional series, and a temporal window of 2011 to 2024) a consistent and accelerating rise in TAVR-SAVR case volume has been documented.

Jawitz and colleagues identified 123 TAVR-SAVR cases in the STS database between July 2011 and March 2015, representing an annual rate that, while small in absolute terms, established the baseline trajectory [29]. By the time of the Hawkins 2023 analysis, which extended through 2021, TAVR-SAVR cases were increasing at a rate of 31.8 to 42.8 cases per year in the national registry, with a linear regression R2 of 0.87 confirming the robustness of this trend [34].

Bowdish and colleagues quantified the growth most starkly, documenting a 4235% overall increase in cardiac surgery after TAVR and an average annual growth rate of 148.1% for TAVR-SAVR specifically over the 2012 to 2023 study period [27]. They further noted that TAVR explant and SAVR accelerated disproportionately after 2019 (the year of low-risk TAVR approval) consistent with the hypothesis that broadening TAVR eligibility to younger, lower-risk patients is seeding a future wave of TAVR valve failures in a population with longer life expectancy.

Fukuhara and colleagues provided the most precise institutional projection, demonstrating a linear increase in TAVR-SAVR from 0% of all aortic valve reoperations in 2011–2012 to 31.3% in 2024, with a regression slope of +4.0% per year (95% CI 3.2–4.7, R2 = 0.97) [28]. Based on linear extrapolation from this single-center trajectory, TAVR-SAVR is projected to surpass SAVR-SAVR in annual volume at approximately 51.4% by 2029. This projection carries inherent uncertainty: it assumes linear growth, draws on data from a single institution, and does not account for potential shifts in practice patterns, device technology, or patient selection. It should therefore be interpreted as a directional estimate rather than a precise forecast. The concordance between the Michigan trajectory and STS national registry volume trends provides some support for the directionality of this projection, though national extrapolation requires caution.

**Figure 2:**
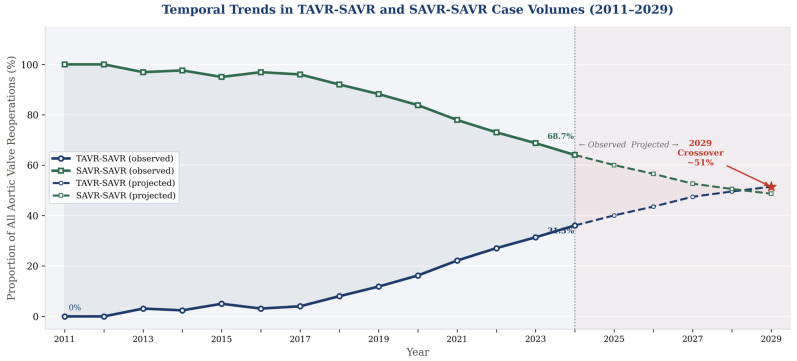
Temporal trends in TAVR-SAVR and SAVR-SAVR case volumes across included registries (2011–2029). Solid lines represent observed data from national and institutional registries; dashed lines represent projected trajectories based on linear regression modeling. The vertical dashed line demarcates observed from projected data (2024). The crossover point at 2029, at which TAVR-SAVR case volume is projected to surpass SAVR-SAVR, is marked with a red indicator. The navy line represents TAVR-SAVR as a percentage of all aortic valve reoperations; the green line represents SAVR-SAVR. Data derived from [19,27,28,34]. Abbreviations: TAVR-SAVR = surgical aortic valve replacement after prior transcatheter aortic valve replacement; SAVR-SAVR = redo surgical aortic valve replacement after prior surgical aortic valve replacement.

The clinical and health systems implications of this trajectory are profound. Centers that currently perform occasional TAVR-SAVR cases will face rapidly growing volumes of a technically demanding, high-risk operation for which standardized expertise, protocols, and outcome benchmarks are still being established. The emergence of TAVR explantation as “the fastest-growing cardiac surgical procedure in the United States” demands an urgent institutional and societal response that prioritizes the development of surgical expertise, multidisciplinary care pathways, and specialized referral networks [27]. The temporal trajectories of TAVR-SAVR and SAVR-SAVR case volumes across the full observation and projection period are illustrated in Figure 2, demonstrating the accelerating divergence between the two pathways and the projected 2029 crossover point at which TAVR-SAVR is expected to surpass SAVR-SAVR as the dominant aortic valve reoperation in the United States.

## 8. Implications for Lifetime Valve Management

The question of optimal initial valve selection for patients with aortic stenosis candidates for both TAVR and SAVR, the so-called “lifetime management” decision, is among the most consequential and contested in contemporary cardiovascular medicine. Current ACC/AHA guidelines recommend transfemoral TAVR as the preferred approach for patients older than 80 years, while endorsing either TAVR or SAVR after shared decision-making for patients aged 65 to 80 years [16]. The 2021 ESC/EACTS guidelines similarly recommend TAVR for patients older than 75 years and high-risk patients, with a class IIa recommendation for SAVR in patients younger than 75 years at low surgical risk [39].

The gray zone of patients aged 65 to 80 years is precisely where the TAVR-SAVR evidence is most clinically relevant. These patients, often with life expectancies of 15 to 25 years, will almost certainly outlive the durability of a transcatheter bioprosthesis implanted today. For them, the choice between TAVR and SAVR is not merely a decision about the current procedure but a decision about the entire sequence of valve interventions they will require over their lifetime. The finding that TAVR-SAVR carries substantially higher operative mortality than SAVR-SAVR, persisting after matching for baseline differences, introduces a previously underquantified cost into the TAVR-first calculus. It must be stated directly, however, that this association does not establish causality. TAVR-SAVR patients are older, have more advanced heart failure, worse renal function, and more frequently present urgently; the observed mortality difference may therefore be fully or partially explained by these patient-level characteristics and the natural trajectory of their disease, rather than by the valve strategy itself. Propensity matching reduces but cannot eliminate this fundamental confounding, and this limitation must remain central to any interpretation of the comparative data.

It is important, however, to interpret this evidence within its appropriate context. The TAVR-SAVR population studied in the available literature is heterogeneous, encompassing patients who underwent TAVR across a range of risk profiles, device generations, and institutional contexts spanning 2011 to 2024. Early-generation devices, higher baseline risk profiles, and limited institutional experience with TAVR explantation in the early years of the study periods likely contribute to the observed mortality figures. As demonstrated by the era-stratified analysis of Fukuhara and colleagues, in which TAVR-SAVR operative mortality declined from 27.3% in 2013–2017 to 5.6% in 2022–2024, outcomes are improving [28]. This temporal trajectory is clinically significant: the aggregate mortality figures cited in this review are weighted toward an earlier era of higher-risk patients and less mature explantation techniques. Contemporary TAVR-SAVR mortality at experienced centers may be substantially lower than the historical composite suggests, and future improvements in patient selection, surgical technique, and perioperative care are likely to further narrow the gap with SAVR-SAVR. The conclusions of this review should therefore be understood as a current snapshot of an evolving landscape rather than a fixed characterization of future risk. The worst historical figures should not be directly extrapolated to contemporary or future patients.

Several additional nuances must be incorporated into lifetime management discussions. First, not all patients who undergo TAVR will require SAVR as their reintervention strategy; ViV-TAVR is feasible and appropriate for many patients with SVD, and ongoing improvements in TAVR repeatability (including the development of technologies to facilitate commissural alignment and coronary access) will expand the proportion of patients for whom redo TAVR is a viable option [40]. The role of ViV-TAVR merits fuller consideration in the lifetime management discussion. Contemporary data from the EXPLANTORREDO-TAVR registry suggest that ViV-TAVR carries lower short-term procedural risk than surgical explantation in selected patients, with its principal limitations being residual hemodynamic impairment (particularly in smaller annuli), coronary obstruction risk, and limited long-term durability data [2,12]. For patients with suitable anatomy and preserved physiological reserve, ViV-TAVR may therefore represent a preferred first reintervention strategy, with surgical explantation reserved for anatomically unsuitable cases or ViV-TAVR failures. Presenting both pathways with appropriate equipoise is essential to balanced lifetime management counseling. Second, the baseline morbidity differences between TAVR-SAVR and SAVR-SAVR patients observed in current studies reflect selection effects that may partially attenuate as TAVR is offered to healthier, lower-risk patients who maintain better biological reserve over time.

In light of the available evidence, several considerations merit attention in clinical practice. First, it may be appropriate for clinicians to discuss with patients aged 65 to 75 years considering TAVR the observation that, should their valve fail and require surgical explantation, available data suggest a substantially higher associated operative mortality compared with redo SAVR after prior surgical prosthesis. This downstream risk information represents one relevant input into shared decision-making, interpreted in the context of its observational origin and the evolving landscape of reintervention options.

Second, preoperative anatomical assessment for future ViV-TAVR feasibility, including coronary height, LVOT dimensions, and prosthesis design compatibility, may be considered at the time of initial TAVR planning, so that patients without suitable anatomy for future transcatheter reintervention can be counseled regarding the implications for their long-term valve management pathway [41].

Third, the available evidence suggests that TAVR explantation may benefit from concentration at high-volume centers with demonstrated explant experience, and that early referral of patients with failing TAVR valves before cardiorenal decompensation becomes irreversible may be associated with improved outcomes. The potential role of dedicated TAVR explant programs, analogous to existing models for complex reoperative cardiac surgery, merits consideration as case volumes continue to grow.

**Figure 3:**
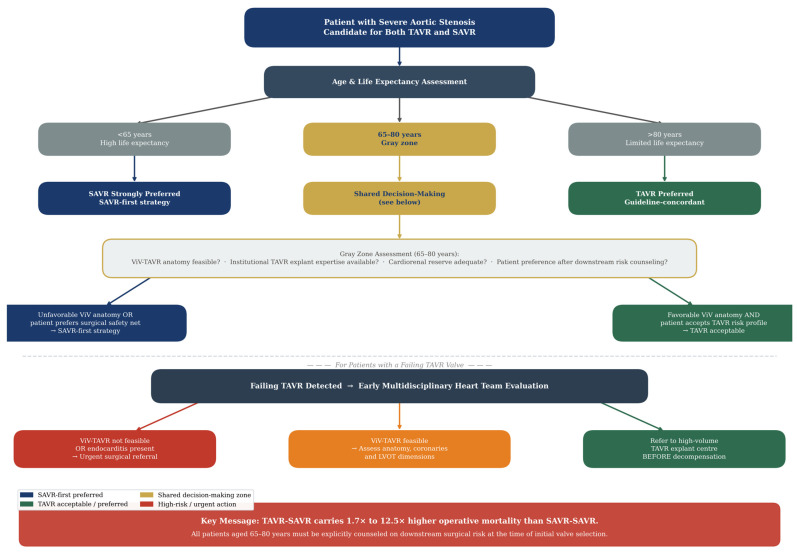
Proposed lifetime valve management decision framework integrating the evidence reviewed. The algorithm stratifies patients by age, surgical risk, aortic root anatomy, and life expectancy to guide initial valve selection between TAVR and SAVR. For patients aged <65 years, SAVR is strongly preferred given the high likelihood of outliving prosthesis durability and the excess mortality associated with TAVR-SAVR should reintervention be required. For patients aged 65–80 years, shared decision-making should explicitly incorporate downstream surgical risk, ViV-TAVR anatomical feasibility, and institutional TAVR explant expertise. For patients aged >80 years, TAVR-first strategies remain appropriate given limited life expectancy relative to prosthesis durability. The framework also specifies criteria for early surgical referral in patients with failing TAVR valves, emphasizing timely intervention before cardiorenal decompensation becomes irreversible. Colours explanation: Purple: SAVR-first preferred; Yellow: Shared decision-making zone; Green: TAVR acceptable/preferred; Red: High-risk/urgent action. Abbreviations: TAVR = transcatheter aortic valve replacement; SAVR = surgical aortic valve replacement; ViV = valve-in-valve; STS-PROM = Society of Thoracic Surgeons Predicted Risk of Mortality.

The REPEAT trial—a multicenter randomized trial comparing redo SAVR to ViV-TAVR for degenerated surgical bioprostheses—will generate important prospective evidence on the comparative outcomes of these two reintervention strategies [42]. However, it does not directly address the question examined in this review (TAVR-SAVR vs. SAVR-SAVR) and will not resolve the lifetime management debate for patients currently choosing between TAVR and SAVR as their initial valve procedure. A proposed lifetime valve management decision framework integrating the evidence reviewed herein is presented in Figure 3, providing a structured clinical algorithm for initial valve selection and reintervention planning stratified by patient age, ViV-TAVR anatomical feasibility, and institutional TAVR explant expertise.

## 9. Limitations of the Current Evidence

Several important limitations of the available evidence must be acknowledged when interpreting the findings of this review. First and most fundamentally, all included studies are retrospective in design. Prospective randomized trials comparing TAVR-SAVR to SAVR-SAVR are not feasible given the clinical realities of the patient population (patients presenting with failed TAVR valves requiring surgical explantation cannot be randomized to a SAVR-SAVR comparator) and the evidence base will therefore remain anchored in observational data for the foreseeable future. Residual confounding, despite sophisticated propensity adjustment, cannot be fully excluded from any observational comparative analysis.

Second, a potential patient-level overlap exists between the two comparative studies. The Hawkins 2023 analysis draws on the STS national database from 2011 to 2021, while the Fukuhara 2026 single-center series covers 2011 to 2024. As the University of Michigan is an STS-participating institution, Michigan patients from 2011 to 2021 are likely included within the Hawkins national cohort. This overlap precludes formal pooling of effect estimates from the two comparative studies, necessitating a narrative synthesis approach.

Third, long-term survival data are not available from any included study. All reported outcomes are restricted to operative or 30-day mortality and in-hospital complications. Whether the mortality excess in TAVR-SAVR attenuates over longer follow-up, as survivors of the perioperative period benefit from a restored functioning aortic valve, or whether it persists through differential rates of late mortality remains unknown.

Fourth, data granularity is limited by registry-based data sources. Key variables including specific TAVR device model, explant technique, time since TAVR implantation, primary valve pathology at the time of initial TAVR, and frailty indices were either unavailable or incompletely reported across studies. These variables may be important confounders or effect modifiers that cannot be adjusted for in current analyses.

## 10. Future Directions

The rapidly evolving landscape of TAVR reintervention demands a commensurate expansion in research activity across several domains. Prospective multicenter registries with standardized data collection (encompassing device model, explant technique, operator volume, multidisciplinary team involvement, and granular preoperative phenotyping including frailty, right ventricular function, and nutritional status) are essential to generate the evidence base necessary for robust comparative analyses and risk model development [38].

The validation and refinement of the STS SAVR-after-TAVR risk calculator represents an urgent priority. While this tool represents a meaningful advance over the standard STS-PROM model for this population, its continued systematic underestimation of operative mortality, particularly in low-risk patients, suggests that additional variables specific to the TAVR-SAVR biological context need to be incorporated. Candidate variables include time since TAVR implantation, right ventricular systolic pressure, hepatic biomarkers of congestion, frailty assessments, and indicators of TAVR-specific technical complexity such as degree of neo-endothelialization [37].

The development and dissemination of standardized TAVR explantation surgical techniques represents another critical research and education priority. While surgical approaches including the double Kocher clamp technique for balloon-expandable valves and the tourniquet technique for self-expanding valves have been described, systematic training programs and competency frameworks for TAVR explantation are absent from current cardiac surgery curricula [36]. Given the projected volume growth documented in this review, the cardiac surgery community faces an urgent educational imperative to develop the next generation of surgeons with proficiency in this increasingly common and technically demanding procedure.

Artificial intelligence and machine learning approaches offer promising avenues for improving lifetime valve management decision-making. Models trained on longitudinal patient data (integrating imaging, genomic, proteomic, and clinical variables) may better capture the multidimensional complexity of lifetime valve trajectory than current single-timepoint risk calculators, potentially enabling personalized prediction of valve durability, reintervention probability, and surgical outcomes for individual patients at the time of initial valve selection [43].

Finally, patient-reported outcomes (quality of life, functional status, and health utility) represent an underexplored dimension of the TAVR-SAVR literature. Survival-focused endpoints, while critically important, do not capture the full patient experience of complex reoperative surgery. Integration of patient-centered outcome measures into future TAVR-SAVR registries will provide a more complete picture of the value proposition of different lifetime management strategies and better inform shared decision-making conversations between clinicians and patients.

## 11. Conclusions

This narrative review of the available comparative evidence demonstrates a consistent association between prior TAVR and significantly higher operative mortality at the time of redo SAVR compared with redo SAVR after prior surgical aortic valve replacement, a pattern that persists after risk adjustment and propensity score matching across two major comparative datasets. Given the observational nature of all included studies and substantial residual baseline differences between groups, a causal attribution of this excess mortality to prior TAVR itself cannot be established with confidence. The observed differences may be fully or substantially explained by patient-level characteristics and disease trajectory rather than the valve strategy per se. These findings are best interpreted as an important signal warranting prospective validation. Observed-to-expected mortality ratios consistently exceed 1.0 in TAVR-SAVR patients across all risk strata, while the SAVR-SAVR pathway at experienced centers achieves a remarkably low O/E ratio of 0.22–0.33. The paradoxical finding that TAVR-SAVR patients have shorter operative times yet worse outcomes provides compelling evidence that excess mortality reflects patient-level biological vulnerability (driven by cumulative cardiorenal injury accumulated over the years between TAVR implantation and reoperation) rather than the technical demands of TAVR explantation itself.

TAVR-SAVR case volumes are growing rapidly and are projected to surpass SAVR-SAVR volumes by 2029. This trajectory demands urgent action across clinical, educational, organizational, and research domains: explicit counseling of patients considering TAVR at ages 65 to 80 years about downstream surgical risk; early referral of patients with failing TAVR valves before cardiorenal decompensation becomes irreversible; development of centers of excellence in TAVR explantation; and investment in risk model validation and surgical training programs commensurate with the projected procedural burden.

The initial valve selection decision in patients with aortic stenosis candidates for both TAVR and SAVR is not merely a choice about the current procedure but a decision about the entire lifetime valve management trajectory and its biological consequences. The available observational evidence consistently demonstrates an association between prior TAVR and higher operative mortality at redo SAVR, persisting after risk adjustment; however, the extent to which this excess reflects the biological impact of prior TAVR itself versus the cumulative comorbidity burden of the TAVR-SAVR population cannot be fully disentangled from retrospective data alone. For operable patients aged 65 to 80 years expected to outlive their prosthesis, these findings highlight the importance of incorporating downstream reintervention risk into the initial valve selection discussion, while acknowledging that rapidly improving outcomes and expanding ViV-TAVR feasibility will continue to reshape this calculus. In the era of expanding TAVR indications, the legacy of the first valve cannot be ignored.

## Figures and Tables

**Table 1:** Characteristics of included studies.

Author, Year	Journal	Database	Period	N TAVR-SAVR	N SAVR-SAVR	Design	Risk Adjustment
Jawitz et al., 2020 [29]	JACC CI	STS Database	2011–2015	123	None (descriptive)	Retrospective registry	O/E ratio only
Hawkins et al., 2023 [34]	JACC CI	STS Database	2011–2021	1126 (PSM: 433)	29,306 (PSM: 433)	Retrospective registry + PSM	Hierarchical regression + 63-variable PSM
Bowdish et al., 2024 [27]	Ann Thorac Surg	STS Database	2012–2023	2972	None (descriptive)	Retrospective registry	Multivariable logistic regression
Fukuhara et al., 2026 [28]	JTCVS	Single-center (Michigan)	2011–2024	127 (PSM: 50)	897 (PSM: 93)	Retrospective single-center + PSM	16-variable PSM; SAVR-after-TAVR STS calculator

Abbreviations: JACC CI = JACC: Cardiovascular Interventions; JTCVS = Journal of Thoracic and Cardiovascular Surgery; STS = Society of Thoracic Surgeons; PSM = propensity score matching; O/E = observed-to-expected; CABG = coronary artery bypass grafting.

**Table 2:** Comparative outcomes across included studies (propensity-matched cohorts where available).

Endpoint	Jawitz 2020 [29] (TAVR-SAVR Only)	Hawkins 2023 [34] TAVR-SAVR PSM	Hawkins 2023 [34] SAVR-SAVR PSM	Fukuhara 2026 [28] TAVR-SAVR PSM	Fukuhara 2026 [28] SAVR-SAVR PSM
Operative mortality	17.1%	11.3% *	6.7%	12.0% **	1.1%
Failure to rescue	—	32% **	16%	—	—
Renal failure	10.4%	9% *	5%	10.4%	3.3%
Prolonged ventilation	40.7%	22%	19%	32.0% ***	9.7%
Hospital LOS (days)	—	8	7	9 **	7
Stroke	3.3%	4%	3%	4.0%	0%
PPM implantation	14.6%	—	—	8.6%	7.1%
O/E mortality ratio	1.49–5.48 †	—	—	1.49	0.22
Composite complication	—	28%	24%	42.0% ***	12.9%
CPB time (min)	146	113 ***	133	131	146

* *p* < 0.05; ** *p* < 0.01; *** *p* < 0.001 vs. matched SAVR-SAVR; † range across risk strata (low, intermediate, high). Abbreviations: PSM = propensity score matching; LOS = length of stay; PPM = permanent pacemaker; O/E = observed-to-expected; CPB = cardiopulmonary bypass; — = not reported.

**Table 3:** Observed-to-expected (O/E) operative mortality ratios by risk stratum across studies.

Study	Overall O/E	Low-Risk O/E (STS-PROM <4%)	Intermediate-Risk O/E (4–8%)	High-Risk O/E (>8%)	Notes
Jawitz 2020 [29] (TAVR-SAVR)	—	5.48 (1.17–13.93)	1.66 (0.35–4.40)	1.16 (0.68–1.79)	Standard STS-PROM; no comparator
Bowdish 2024 [27] (TAVR-SAVR, AVR subcohort)	1.42	—	—	—	STS standard model; procedure-specific subcohorts
Fukuhara 2026 [28] (TAVR-SAVR, pre-match)	1.50 (0.60–3.09)	1.80	1.58	1.21	SAVR-after-TAVR STS calculator; stratified by index TAVR risk
Fukuhara 2026 [28] (SAVR-SAVR, pre-match)	0.33 (0.11–0.76)	—	—	—	Standard STS-PROM; experienced center
Fukuhara 2026 [28] (TAVR-SAVR, PSM)	1.49 (0.54–3.24)	—	—	—	SAVR-after-TAVR calculator; matched cohort

Abbreviations: STS-PROM = Society of Thoracic Surgeons Predicted Risk of Mortality; AVR = aortic valve replacement; PSM = propensity score matching; O/E = observed-to-expected; — = not reported or not applicable. Values in parentheses represent 95% confidence intervals where reported.

## Data Availability

The data that support the findings of this study are available from the corresponding author, upon reasonable request.

## Abbreviations

The following abbreviations are used in this manuscript:

ACC/AHA	American College of Cardiology/American Heart Association
AVR	Aortic valve replacement
CABG	Coronary artery bypass grafting
CPB	Cardiopulmonary bypass
ESC/EACTS	European Society of Cardiology/European Association for Cardio-Thoracic Surgery
ICU	Intensive care unit
JACC CI	JACC: Cardiovascular Interventions
JTCVS	Journal of Thoracic and Cardiovascular Surgery
LOS	Length of stay
LVOT	Left ventricular outflow tract
MVR	Mitral valve replacement
NOTION	Nordic Aortic Valve Intervention trial
O/E	Observed-to-expected
OR	Odds ratio
PARTNER	Placement of Aortic Transcatheter Valves trial
PPM	Permanent pacemaker implantation
PSM	Propensity score matching
PVE	Prosthetic valve endocarditis
PVL	Paravalvular leak
REPEAT	Redo surgical aortic valve replacement versus valve-in-valve transcatheter aortic valve replacement trial
SAVR	Surgical aortic valve replacement
SAVR-SAVR	Redo surgical aortic valve replacement after prior surgical aortic valve replacement
SAVR-TAVR-SAVR	Redo surgical aortic valve replacement after prior surgical aortic valve replacement and subsequent transcatheter aortic valve replacement
STS	Society of Thoracic Surgeons
STS-PROM	Society of Thoracic Surgeons Predicted Risk of Mortality
SVD	Structural valve deterioration
TAVR	Transcatheter aortic valve replacement
TAVR-SAVR	Surgical aortic valve replacement after prior transcatheter aortic valve replacement
ViV-TAVR	Valve-in-valve transcatheter aortic valve replacement
